# Supplementary figures and images for: Ceramide-1-phosphate transfer protein enhances lipid transport by disrupting hydrophobic lipid–membrane contacts
Source: PLoS Comput Biol. 2023 Apr 10;19(4):e1010992. doi: 10.1371/journal.pcbi.1010992 (PMC10085062; doi:10.1371/journal.pcbi.1010992)

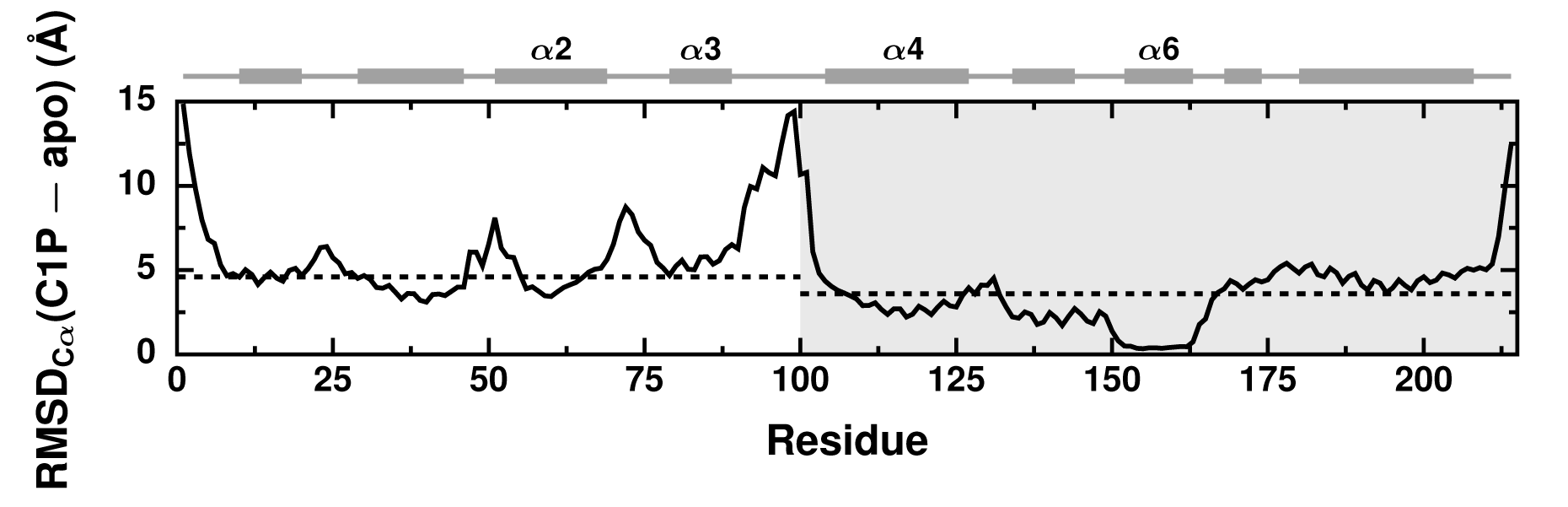

Supplement: S1 Fig — Root-mean-square deviation (RMSD) of each residue’s Cα atom of CPTP in its apo form relative to its C1P-bound form during solution-phase all-atom simulations. After alignment of the structures to minimize the RMSD of Cα atoms of helix α6, the RMSD between each Cα in the C1P-bound and apo forms is calculated as RMSDCα(C1P-apo)=t-2∑ij(rC1P(ti)-rapo(tj))2, where i and j index all t frames in each trajectory and rC1P(t) and rapo(t) are the Cα’s positions in the C1P-bound and apo forms, respectively. CPTP’s secondary structure is schematically illustrated above with helices represented as rectangles and unstructured loop regions as lines. The gray region highlights residues that comprise one side of CPTP’s sandwich-like structure. The dashed lines indicated the average RMSDCα(C1P − apo) for helices αN and α1 − 3 of 4.6 Å (white region) and helices α4, α5, α7, and α8 of 3.6 Å (gray region). (TIF) [file pcbi.1010992.s001.tif]

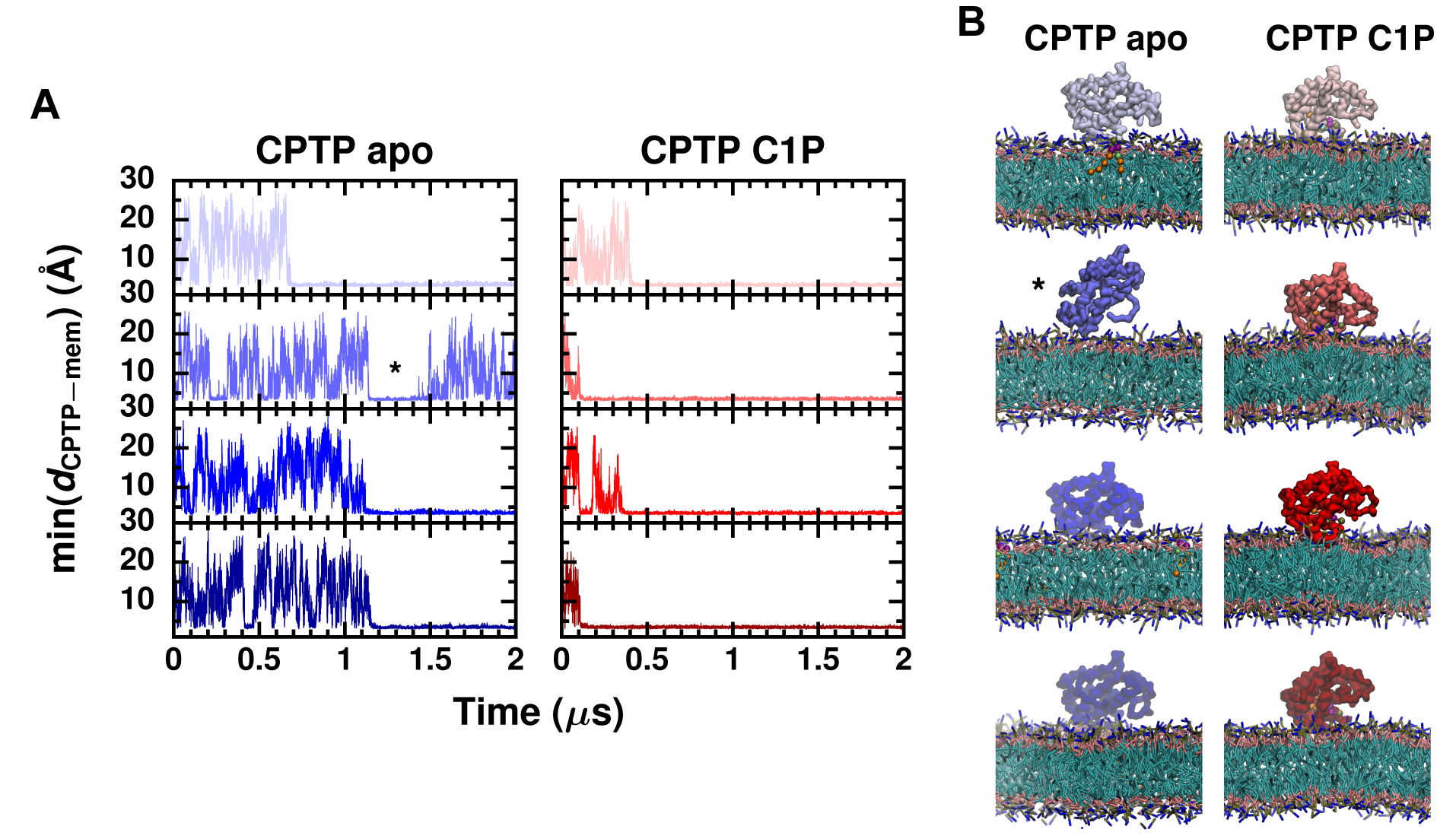

Supplement: S2 Fig — (A) Minimum distance between CPTP and the membrane during coarse-grained simulations of membrane binding. (B) Configurations observed at the end of each simulation. Four independent simulations were performed for each form of CPTP. Within 2 μs, CPTP stably bound the membrane in all but one simulation of the apo form (indicated with an asterisk). (TIF) [file pcbi.1010992.s002.tif]

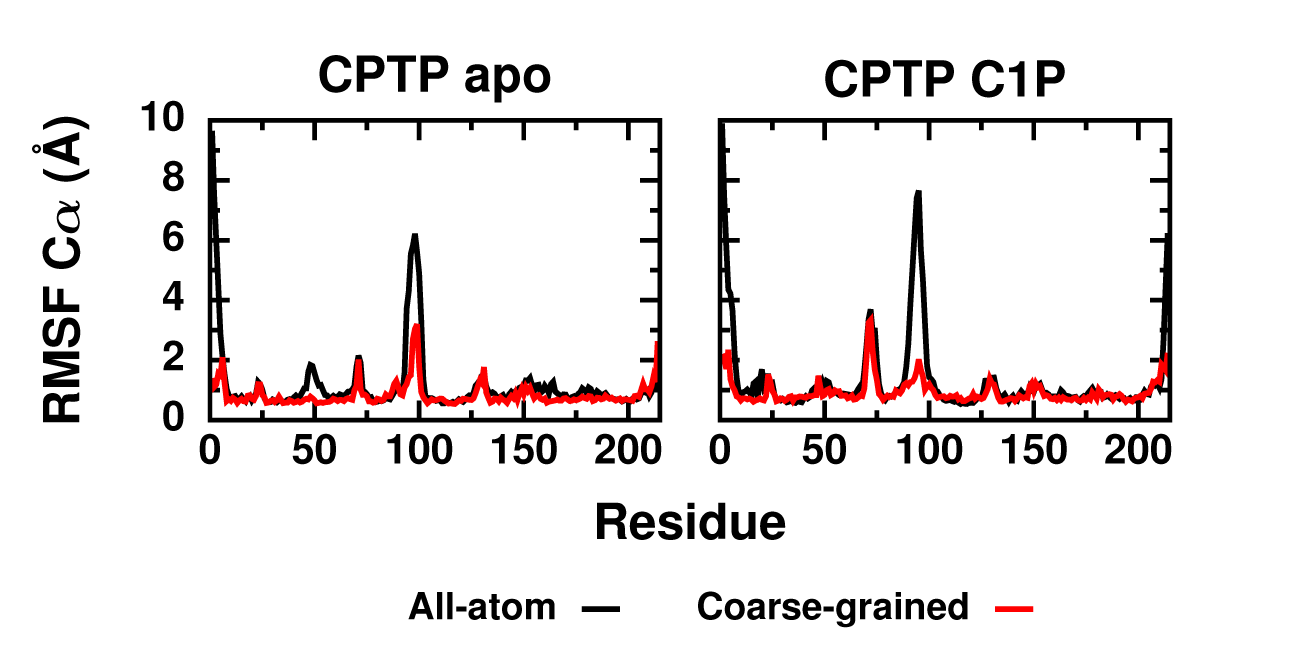

Supplement: S3 Fig — Root-mean-square fluctuation (RMSF) of Cα atoms (backbone beads) of CPTP in its apo and C1P-bound forms during solution-phase all-atom (coarse-grained) simulations. (TIF) [file pcbi.1010992.s003.tif]

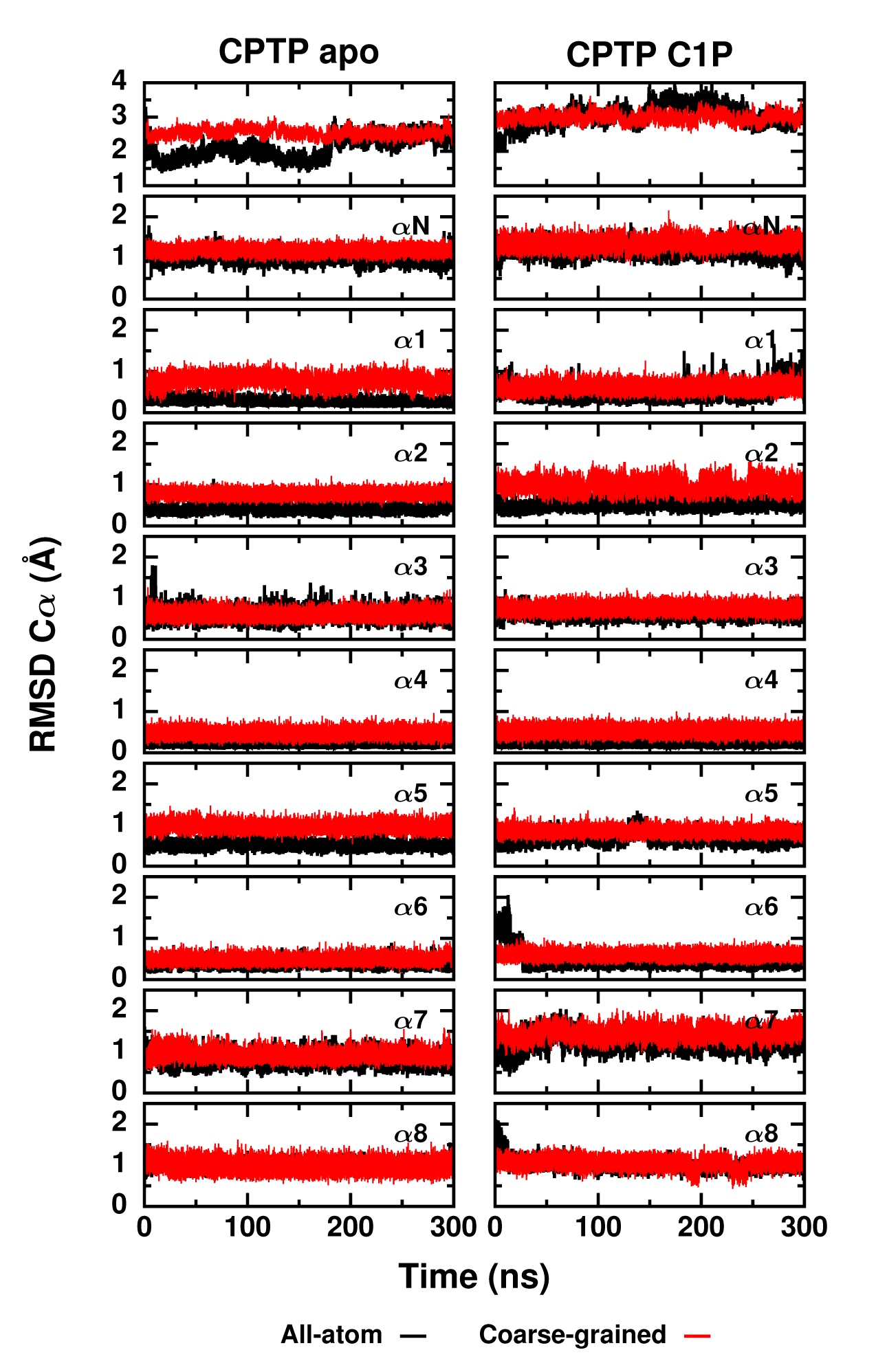

Supplement: S4 Fig — Root-mean-square deviation (RMSD) of Cα atoms (backbone beads) between CPTP in its apo and C1P-bound forms during solution-phase all-atom (coarse-grained) simulations and the cystral structure of CPTP in PDB 4K85. In the top row, the RMSD for residues 8 − 214 is plotted. All other rows show the RMSD for individual helices. (TIF) [file pcbi.1010992.s004.tif]

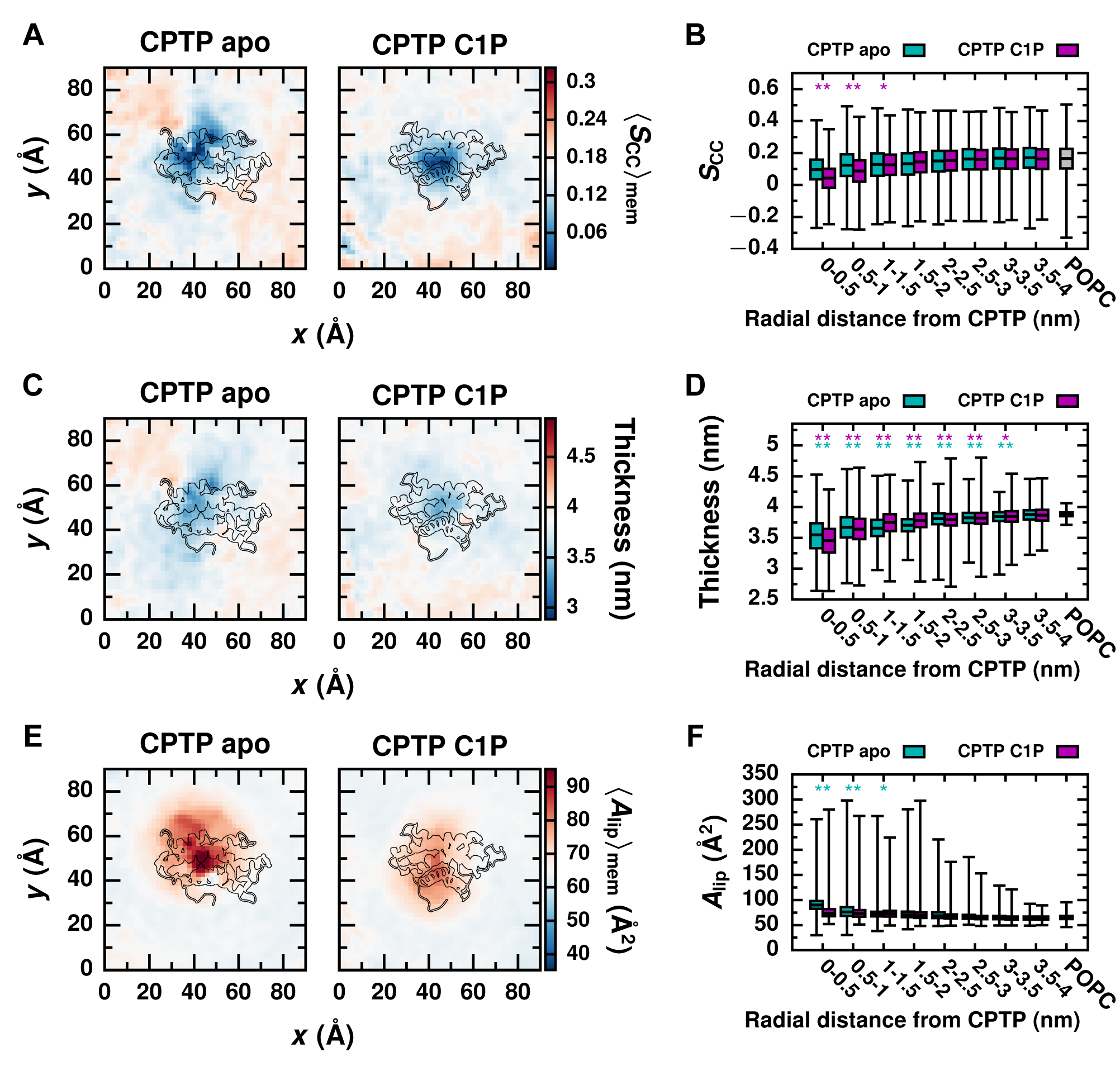

Supplement: S5 Fig — CPTP impacts (A and B) the average orientational order parameter of lipids’ acyl chains, SCC [84], (C and D) membrane thickness, and (E and F) area per lipid. (A, C, and E) Average of each property as a function of a lipid’s displacement in the xy plane from CPTP. Color scales are set relative to the average value for a POPC membrane without CPTP present (white). Structures of the apo and C1P-bound forms of CPTP bound to the membrane are outlined. (B, D, and F) Box-and-whisker plots of each property for lipids within a specified radial distance from CPTP’s center-of-mass in the xy plane and for lipids in a POPC membrane without CPTP present. The box extends from the 1st to 3rd quartile with the median indicated by the black line, and the whiskers extend from the minimum to maximum value. Asterisks indicate the average differs significantly from that of a POPC membrane: ** p < 0.001; * p < 0.01 (Welch’s t-test). (TIF) [file pcbi.1010992.s005.tif]

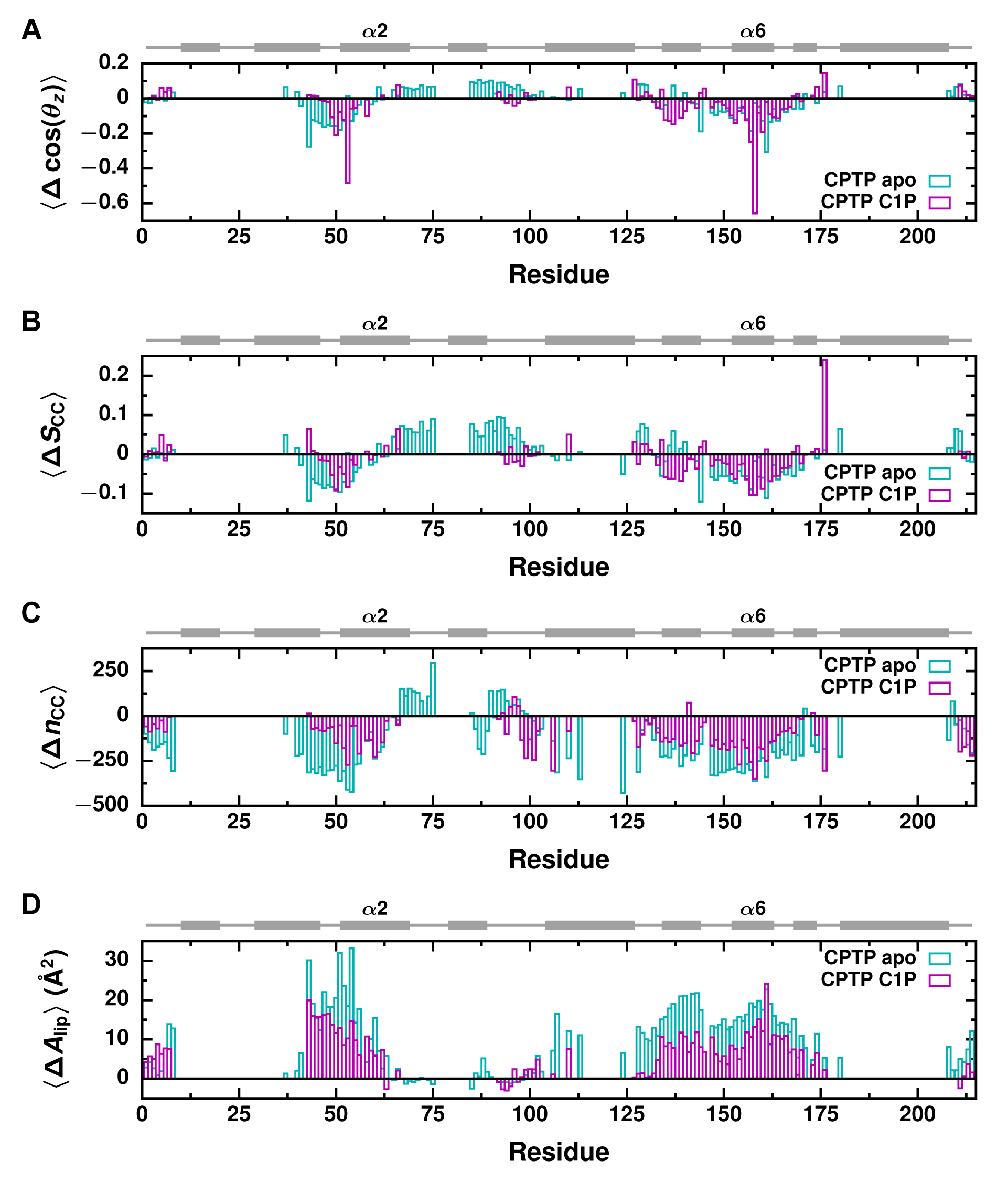

Supplement: S6 Fig — In both the apo and C1P-bound forms, residues on helices α2 and α6 promote lipid reorientation. Residues on helix α5 of the C1P-bound form promote further increases in lipid reorientation and disorder. Residues on helices α1, α2, and α6, especially in the the apo form of CPTP, promote decreases in local membrane hydrophobicity and increases in the area per lipid. Average change in (A) cos(θz) (Fig 5A), (B) SCC [84], (C) nCC (Fig 6A), and (D) area per lipid, Alip, relative to the average of a POPC membrane without CPTP present for lipids within 5Å of each residue of CPTP. CPTP’s secondary structure is schematically illustrated above with helices represented as rectangles and unstructured loop regions as lines. (TIF) [file pcbi.1010992.s006.tif]

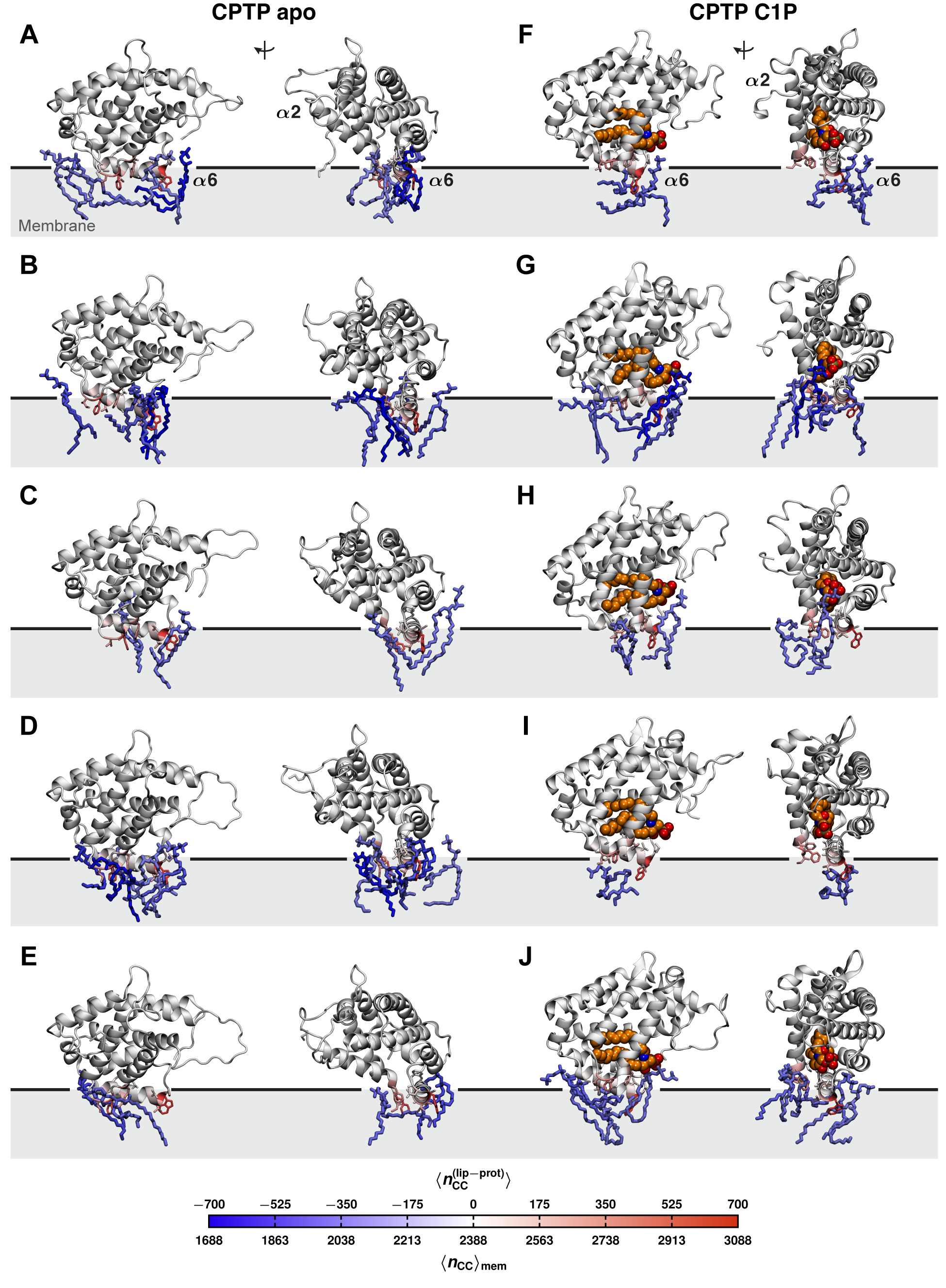

Supplement: S7 Fig — 〈nCC(lip-prot)〉 mapped onto the structures of the (A-E) apo and (F-J) C1P-bound forms. Example configurations of lipids with (1) a reduction in nCC of ≥ 300 contacts relative to 〈nCC〉mem of a POPC membrane without CPTP bound and (2) ≥ 90% of those contacts replaced with hydrophobic contacts with CPTP are shown. The black line indicates the average position of phosphate groups of membrane lipids. (TIF) [file pcbi.1010992.s007.tif]

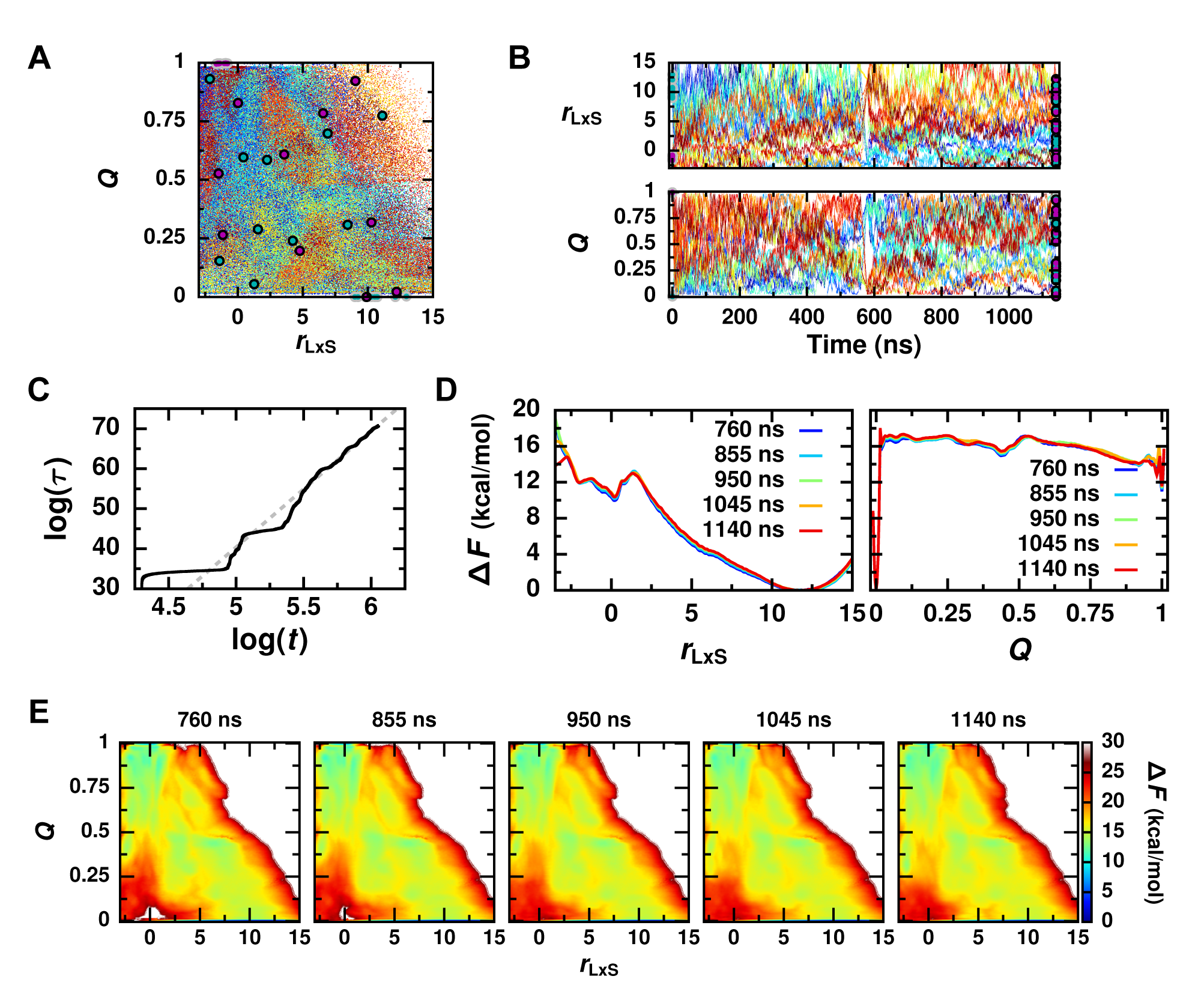

Supplement: S8 Fig — (A and B) Walkers initiated from the apo (cyan points outlined in gray) and C1P-bound forms (magenta points outlined in gray) broadly and diffusively sample the space of rLxS and Q and are well-mixed by the end of the simulation. Values of rLxS and Q at the end of the simulation are indicated with points outlined in black for each walker (cyan for those initialized from the apo form and magenta for those initialized from the C1P-bound form). (A) Values of rLxS and Q sampled during the simulation. Each point is colored by time with configurations sampled at early times shown in dark blue and at later times in dark red. (B) Trajectories of rLxS and Q for each walker. Walkers initiated from the apo form are plotted in cool colors, and walkers initiated from the C1P-bound form are plotted in warm colors. (C) Scaled time computed from the time-dependent bias is plotted versus simulation time on a log-log scale. The dashed gray line is the linear fit after 500 ns. The slope of this line is 29, which is consistent with the bias factor of 30 used in the simulation and suggests that a quasi-steady state has been reached. (D and E) Agreement between free energy profiles calculated at simulation times ranging from 760 ns (cumulatively 15.2 μs) to 1140 ns (cumulatively 22.8 μs) indicates convergence. All free energy profiles were calculated by reweighting frames after 500 ns (cumulatively 10 μs) up to the specified time using the estimator of Tiwary and Parrinello [91]. (TIF) [file pcbi.1010992.s008.tif]

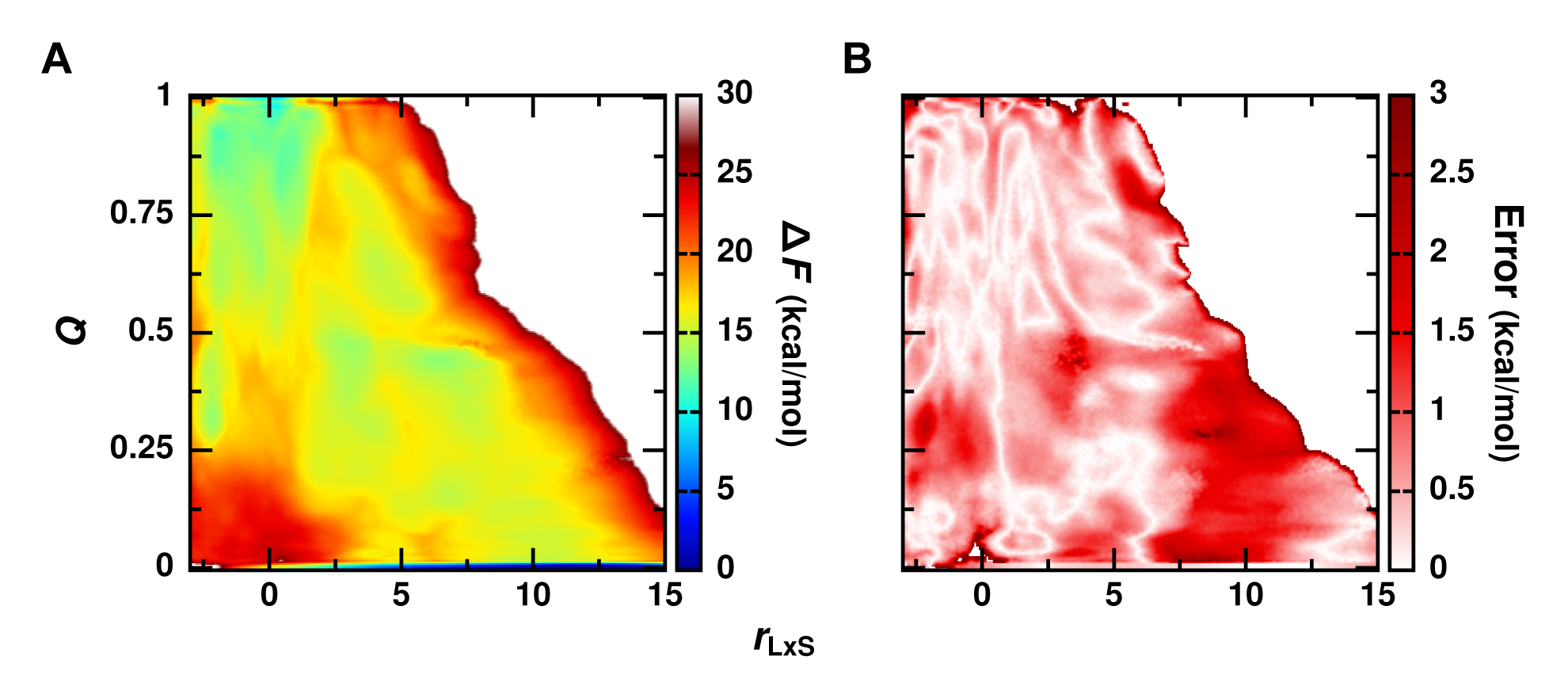

Supplement: S9 Fig — (A) The free energy surface ΔF(rLxS, Q) shown in Fig 7A is reproduced. (B) Standard error of ΔF(rLxS, Q) computed with block averaging. (TIF) [file pcbi.1010992.s009.tif]

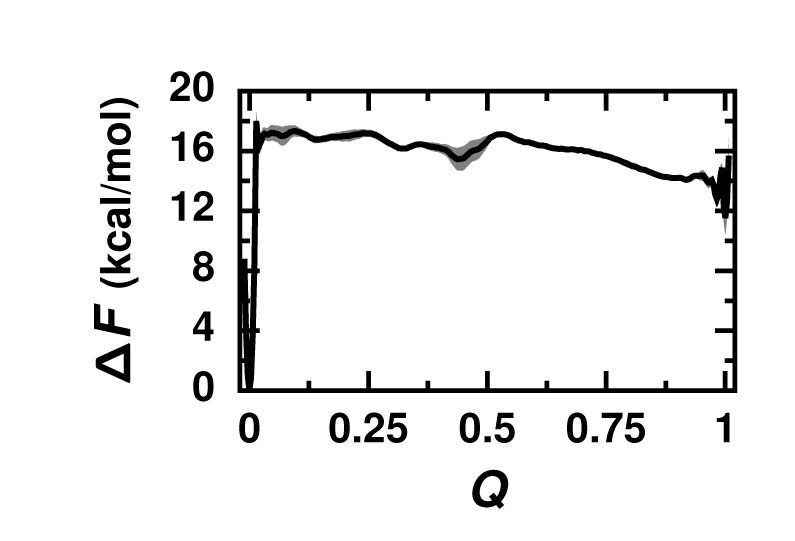

Supplement: S10 Fig — ΔF(Q) is obtained by marginalizing ΔF(rLxS, Q) over rLxS. Error bars indicate the standard error computed with block averaging. (TIF) [file pcbi.1010992.s010.tif]

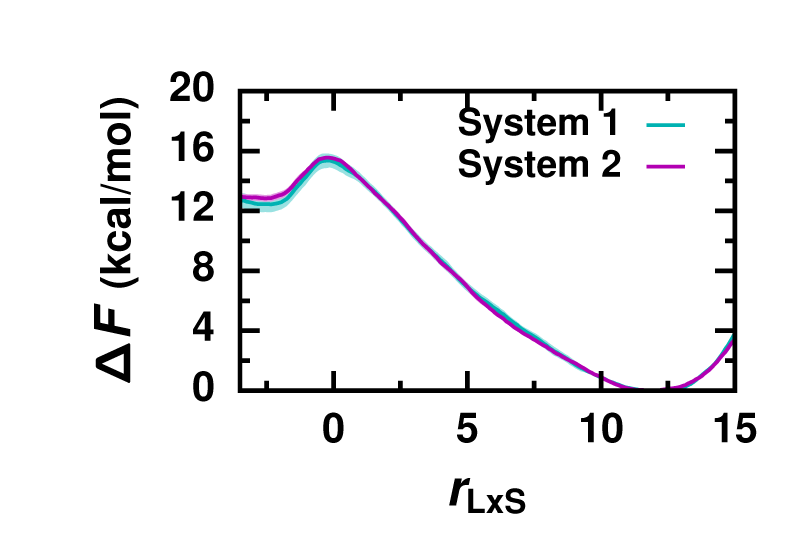

Supplement: S11 Fig — Free energy profiles along rLxS calculated for two independent systems. For each system’s free energy profile, error bars were calculated as the standard error of ΔF estimated from four independent 8 ns blocks. (TIF) [file pcbi.1010992.s011.tif]

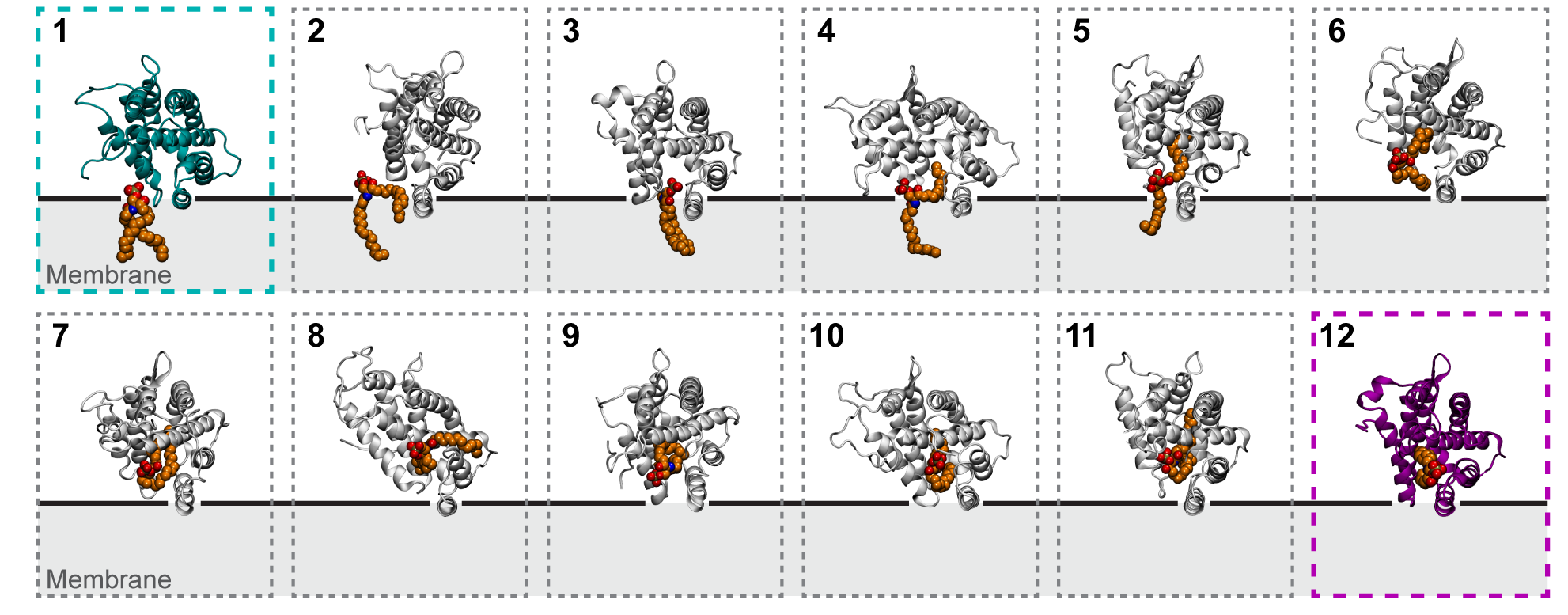

Supplement: S12 Fig — View looking into CPTP’s hydrophobic cavity of example configurations from different regions of the free energy surface outlined in Fig 7A. C1P is rendered as van der Waals spheres and colored orange. The apo form of CPTP in configuration 1 is colored dark cyan, and the C1P-bound form in configuration 12 is colored dark magenta. The black line indicates the average position of phosphate groups of membrane lipids. (TIF) [file pcbi.1010992.s012.tif]
