# Supplementary material for: Ceramide-1-phosphate transfer protein enhances lipid transport by disrupting hydrophobic lipid–membrane contacts
Source: PLoS Comput Biol. 2023 Apr 10;19(4):e1010992. doi: 10.1371/journal.pcbi.1010992 (PMC10085062; doi:10.1371/journal.pcbi.1010992)
Supplement: S1 Text — Discussion of (A) conformational differences between apo and C1P-bound forms of CPTP both in solution-phase and membrane-bound simulations analyzed with principal component analysis and (B) choice of C1P–CPTP contacts used to define Q. (PDF) [file pcbi.1010992.s013.pdf]

SUPPORTING NOTES

Note A: Principal component analysis of CPTP configurations. We additionally characterize differences in the all-atom configurations of the apo and C1P-bound forms of CPTP both in solution and bound to the membrane through principal component analysis (PCA). Specifically, PCA was performed on frames from all simulations after alignment about Cα atoms of helix α6, which approximates the plane of the membrane surface, to characterize variations in the Cartesian coordinates of helices' backbone atoms. PCA was performed using GROMACS tools.

The first principal component (PC1) captures 48% of the total variation in the structures, and PCs 1-4 collectively capture 89% of the total variance (Fig A panel A). Fig A panels B-D illustrate the structural

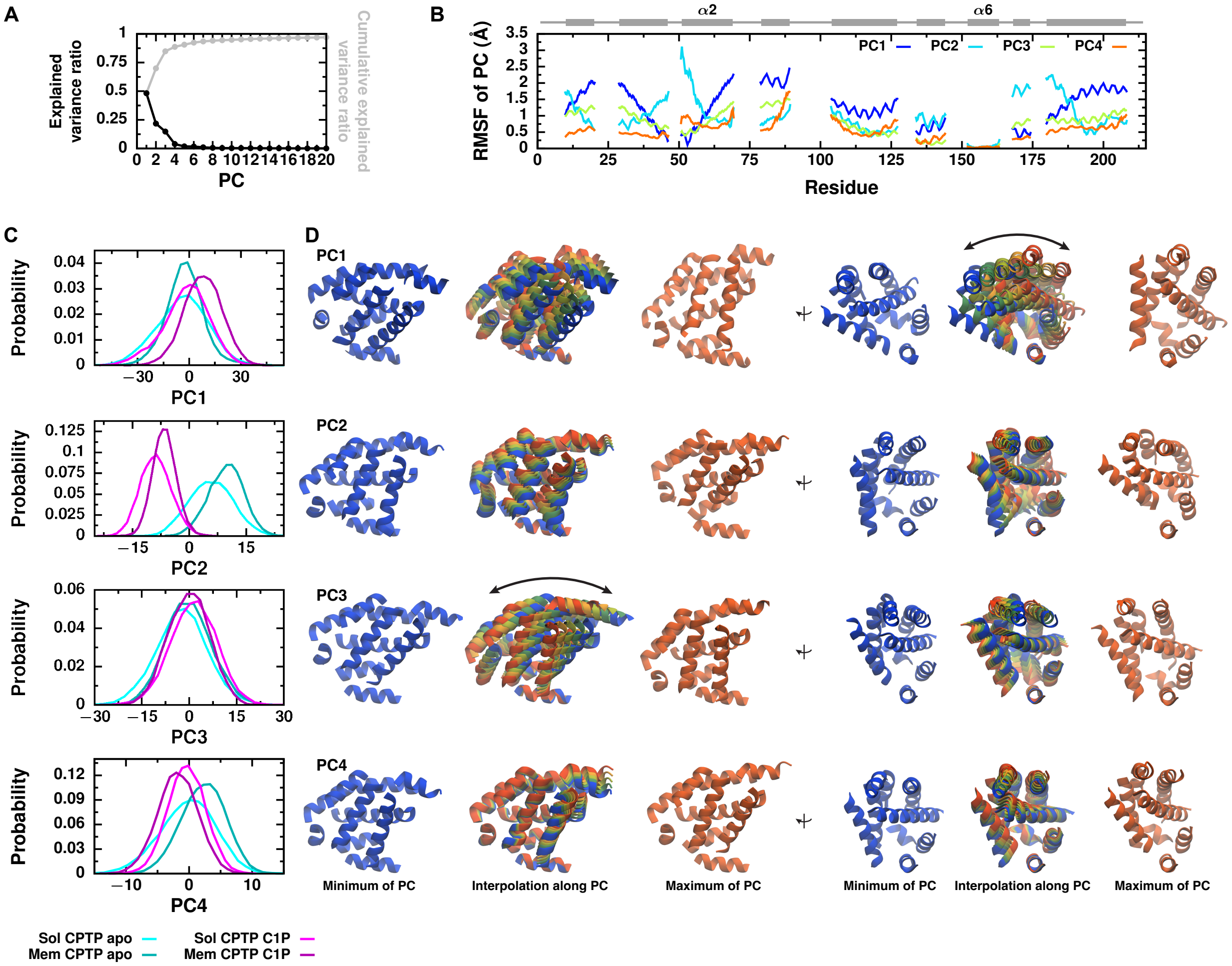

Fig A. Principal component analysis (PCA) captures differences in the all-atom structures of the apo and C1P-bound forms of CPTP both in solution and bound to the membrane. PCA was performed on helices' backbone atoms' Cartesian coordinates after alignment about the Cα atoms of helix α6. (A) Explained variance ratio of each PC and cumulative explained variance ratio. (B) Root-mean-square fluctuation (RMSF) of each atom along PCs 1-4. CPTP's secondary structure is schematically illustrated above with helices represented as rectangles and unstructured loop regions as lines. (C) Configurations were projected on each PC, and distributions of the PC values are shown for the apo and C1P-bound forms of CPTP both in solution-phase and membrane-bound simulations. (D) Structural differences captured by each PC are illustrated by structural interpolations between the extreme projections along each PC.

differences described by PCs 1-4 and the distributions of PC values sampled in solution-phase and membrane-bound simulations of both apo and C1P-bound forms of CPTP:

PC1 describes a concerted rotation about helix $\alpha 6$, which approximates the surface of the membrane. Negative values of PC1 correspond to structures in which helix $\alpha 2$ is located closer and more parallel to the membrane surface, whereas positive values correspond to structures with helix $\alpha 2$ orientated more perpendicular to the membrane surface. We find that structures of the C1P-bound form of CPTP bound to the membrane have increased values of PC1 on average compared to either the C1P-bound form in solution or the apo form. Thus, membrane binding of the C1P-bound form promotes a concerted reorientation that aids the opening of gating helix $\alpha 2$ and that positions a widened entrance to CPTP's hydrophobic cavity at the membrane surface.

PC2 describes an internal reorganization of CPTP's helices that rotates the sides of its sandwich-like structure relative to each other (as if two stacked planar sheets were rotated relative to each other). Such changes captured by PC2 are evocative of a cleft-like gating mechanism (Fig 1 and S1 Fig) [16]. Of the first four PCs, variation along PC2 best captures differences between the apo and C1P-bound forms of CPTP, regardless of if CPTP is in solution or bound to the membrane. Indeed, C1P uptake (or release) results in substantial rearrangement of the sides of CPTP's sandwich-like structure relative to each other (Fig 1 and S1 Fig). Membrane-bound structures of both the apo and C1P-bound forms of CPTP have increased values of PC2 on average compared to their respective solution-phase structures. Thus, membrane binding promotes a consistent change in the cleft to CPTP's hydrophobic cavity.

PC3 describes a concerted rotation orthogonal to that of PC1; if motion along PC1 were described as 'rocking side-to-side', then motion along PC3 would be described as 'rocking forward-and-backward'. Conformational ensembles of the apo and C1P-bound forms sampled in both solution-phase and membrane-bound simulations exhibit similar variation along PC3.

PC4 describes an internal reorganization of CPTP's helices different from that of PC2. While solution-phase structures of CPTP have similar average values of PC4, the membrane-bound structures of the apo and C1P-bound forms have average values different from each other and from the solution-phase structures.

Overall, PCA indicates that structures of the apo and C1P-bound forms differ both in solution and bound to the membrane, and that membrane binding can promote opening of gating helix $\alpha 2$ and conformational changes suggestive of a cleft-like gating mechanism.

Note B: Definition of Q. The fraction of contacts C1P makes with CPTP when fully inside its hydrophobic cavity, Q, was used as a second order parameter (or collective variable) for biased simulations. Q was chosen to reliably identify configurations with C1P inside CPTP versus configurations with C1P outside CPTP and to enhance the sampling of CPTP–C1P interactions. Since r_{LxS} only describes C1P–membrane interactions, it accomplishes neither of these things, while Q does. Specifically, CPTP–C1P contact pairs used to calculate Q were selected to capture:

1. Hydrophobic contacts between carbons of C1P and carbons of residues lining CPTP's hydrophobic cavity. Carbon-carbon (CC) pairs were selected based on their average distance, $d_{\mathrm{C1P-CPTP}}$, in solution-phase simulations of the C1P-bound form of CPTP (Fig B panel A). To minimize the computational expensive of calculating Q during biased simulations, which increases with the number of CC pairs considered, while accurately identifying configurations with C1P inside CPTP's hydrophobic cavity, a cutoff of $d_{\mathrm{C1P-CPTP}} \leq 7.8$ Å was used to select these CC pairs. Residues with these CC pairs used to calculate Q are shown in Fig B panel C.

2. Polar contacts between C1P's headgroup and sphingoid backbone and residues at the entrance to CPTP's hydrophobic cavity. Heavy atom pairs were selected based on $d_{\mathrm{C1P-CPTP}}$ (Fig B panel B). To minimize the computational expense of calculating Q during biased simulations while capturing all important polar contacts, a cutoff of $d_{\mathrm{C1P-CPTP}} \leq 5.5$ Å was used to select these pairs. Residues with these polar atom pairs used to calculate Q are shown in Fig B panel D.

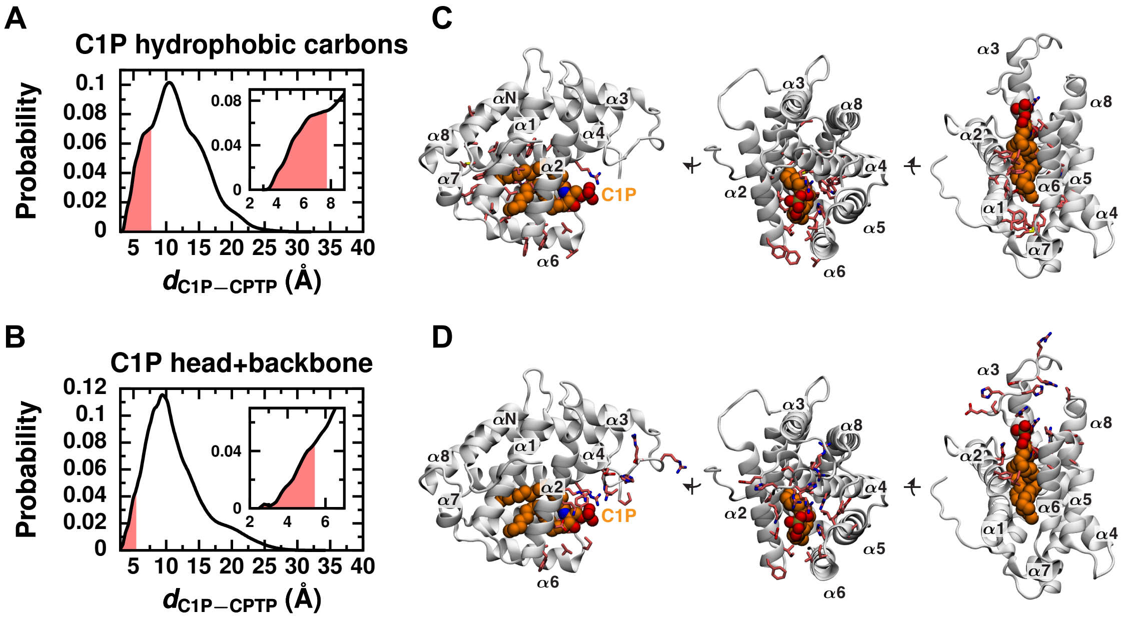

Fig B. CPTP–C1P contacts used to define Q account for key hydrophobic and polar interactions. (A and B) Distributions of the average distance, $d_{\mathrm{C1P-CPTP}}$, between (A) carbon-carbon pairs and (B) pairs of polar atoms of C1P and CPTP during all-atom solution-phase simulations of the C1P-bound form of CPTP. Carbon-carbon pairs with $d_{\mathrm{C1P-CPTP}} \leq 7.8$ Å are used to calculate Q and highlighted by the red region in (A). Residues with these carbon-carbon pairs are rendered in licorice and colored red in (C). Pairs of polar atoms with $d_{\mathrm{C1P-CPTP}} \leq 5.5$ Å are used to calculate Q and highlighted by the red region in (B). Residues with these pairs of polar atoms are rendered in licorice and colored red in (D).

Based on these criteria for selecting contact pairs, 1,176 atom pairs are used to calculate Q using Eq. 4 given in the Methods.

We confirmed that Q reliably identifies configurations with C1P fully inside CPTP's hydrophobic cavity and orientated as in crystal structures from configurations with C1P outside, partially inside, or improperly

orientated within CPTP's cavity. To do so, we monitored the value of Q during all-atom simulations in which C1P enters into CPTP's hydrophobic cavity from the solvent. While the relaxation process that occurs during these simulations is not cellularly relevant, it is sufficiently rapid to observe in unbiased simulations. Thus, we are able to harvest multiple all-atom trajectories of C1P entry from solvent and use them to benchmark Q. Five simulations, each initialized with C1P randomly placed in the solvent around the apo form of CPTP, were performed using the same parameters as the all-atom solution-phase simulations described in the Methods. Each simulation was run for a maximum of 2 μs or until both tails of C1P were inserted into CPTP and no longer exposed to solvent based on visual inspection.

Fig C shows the value of Q during each of these simulations and the final configuration of C1P bound to CPTP. In three of the simulations (shown in green, cyan, and blue in Fig C), C1P inserts between the seam

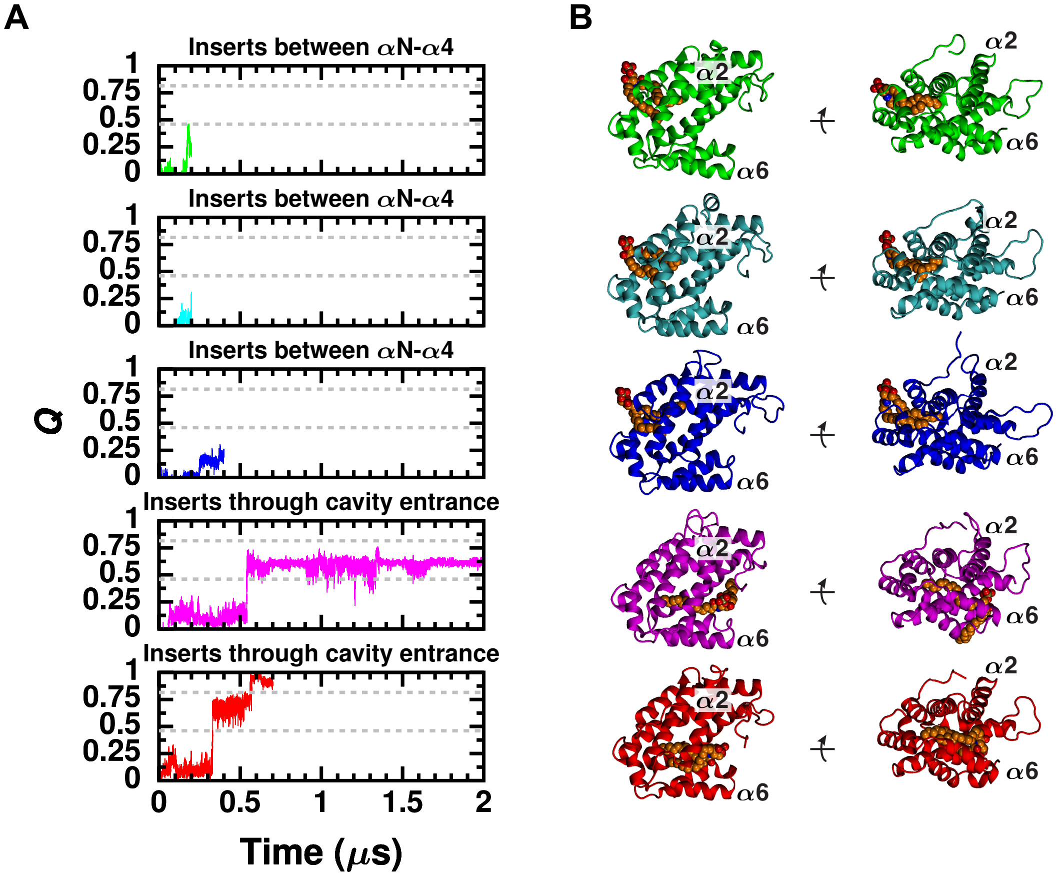

Fig C. Analysis of unbiased simulations of C1P entry into CPTP's hydrophobic cavity from solvent indicate that Q reliably distinguishes configurations with C1P fully inside CPTP's hydrophobic cavity from others. (A) Value of Q during five independent simulations of C1P entry from solvent. In the green, cyan, and blue trajectories, C1P enters through the top seam between helices αN and α4. In the magenta and red trajectories, C1P enters through the entrance identified in crystal structures and located at the membrane surface in simulations. The dashed lines mark the values of Q where sharp transitions occur in the red trajectory. (B) Final configurations of each simulated trajectory.

created by helices αN and α4. These trajectories are not representative of C1P uptake from a membrane since insertion through helices αN and α4, which are fully exposed to solvent when CPTP is bound to a membrane (Fig 2), would require C1P to become fully solvated before entering CPTP's hydrophobic cavity. Thus, they serve as valuable tests of using Q to accurately identify configurations with C1P properly housed in CPTP's cavity. $Q = 1$ when C1P is properly housed inside CPTP's cavity, whereas Q never surpasses 0.5 in these trajectories. In the other two trajectories (shown in magenta and red in Fig C), C1P inserts through the entrance to CPTP's hydrophobic cavity as occurs when it's extracted from a membrane. In

both trajectories, C1P's tails enter individually. In the trajectory shown in magenta in Fig C, the second tail fails to enter within 2 μs. In the trajectory shown in red in Fig C, both tails enter within 700 ns. In both trajectories, Q rapidly changes from $Q \approx 0.1$ to $Q \approx 0.6$ when the first tail enters, and, in the red trajectory, then changes rapidly again when the second tail enters. Thus, Q distinguishes different ways that C1P can bind to CPTP and can be used to enhance the sampling of interactions between C1P and CPTP. We note that these trajectories do not provide any evidence that Q is the reaction coordinate [32–34] (or necessarily a component of the reaction coordinate) for CPTP-mediated C1P transport.
